# Attendance Among People With Hepatitis B in UK Secondary Care: A Scoping Review

**DOI:** 10.1111/jvh.70221

**Published:** 2026-08-12

**Authors:** Emma Victoria Shiel, Bini George, Arpana Verma, Norina Gasteiger

**Affiliations:** ^1^ Mental Health Research Group, Division of Nursing, Midwifery and Social Work, Faculty of Biology, Medicine, and Health University of Manchester Manchester UK; ^2^ Manchester University NHS Foundation Trust Manchester UK; ^3^ School of Medical Sciences, Faculty of Biology, Medicine, and Health University of Manchester Manchester UK; ^4^ Division of Nursing, Midwifery and Social Work, Faculty of Biology, Medicine, and Health University of Manchester Manchester UK

**Keywords:** health equity, Hepatitis B, non attendance, no‐show patients, scoping review, secondary health care

## Abstract

Chronic Hepatitis B disease requires lifelong monitoring to prevent complications such as cirrhosis and hepatocellular carcinoma. Despite structured care pathways, non‐attendance within UK secondary care remains challenging. This scoping review aimed to synthesise existing evidence on factors influencing non‐attendance at secondary care appointments and strategies to improve appointment adherence among people living with Chronic Hepatitis B. Eligibility Criteria: Peer‐reviewed sources examining adults with Hepatitis B attendance at UK secondary care services. Grey literature was excluded. Sources of Evidence: Searches were performed in CINAHL Ultimate, Medline, Embase, Scopus and APA PsycINFO. Data were charted using a structured data extraction form and synthesised narratively. Eleven studies met the inclusion criteria. Identified barriers included patient‐related factors (e.g., limited health literacy, stigmatisation, competing priorities), system‐level challenges (e.g., appointment scheduling and accessibility) and cultural or language barriers. Psychological factors, including fear of diagnosis and treatment side effects, were also reported. Reported strategies included patient education, culturally tailored interventions, reminder systems (e.g., SMS and telephone calls) and integrated care models involving community outreach. Non‐attendance among people with chronic Hepatitis B in secondary care is influenced by a complex interplay of personal, social and systemic factors. Interventions that address multiple barriers, particularly those that incorporate culturally sensitive approaches and reminder systems, may improve engagement. Further research is needed to co‐design and evaluate interventions with affected communities.

## 1 Introduction

Hepatitis B is a major global health concern requiring ongoing monitoring and long‐term management [1]. Chronic Hepatitis B infection can lead to liver cirrhosis and increases the likelihood of developing hepatocellular carcinoma [2]. Attendance at secondary care clinic appointments is therefore crucial, as these appointments provide specialist follow‐up to monitor disease progression and treatment response. The World Health Organization (WHO) has set a target to eliminate Hepatitis B as a public health threat by 2030 [3, 4], however, substantial support is needed to ensure that 80% of the affected population receives treatment by this timeframe [5]. A key challenge to meeting the target is appointment non‐attendance, which is common (an average of 23%) and disrupts continuity of care, increases waiting times, and negatively affects patient outcomes and the efficiency of the National Health Service (NHS) [6, 7, 8]. Treatment adherence is also inconsistent, ranging from 15% to over 70% [9]. Personalised care plans are therefore recommended by the United Kingdom's (UK) National Institute for Health and Care Excellence, as encouraging patients to take an active role in their care is expected to improve adherence to monitoring, treatment and screening/tests and minimise non‐attendance [10].

Because Hepatitis B treatment tends to be long‐term or indefinite, some attendance issues are expected [9]. However, literature specific to Hepatitis B shows associations with patient demographics (males being more likely to no‐show), seasonal variation, health status, appointment type (e.g., whether they are face‐to‐face or telephone), patient‐related behaviours (e.g., motivation or interest in health) and socioeconomic barriers [11, 12, 13], indicating potential inequities. The broader hepatology literature also highlights additional contributors, such as language difficulties, long waiting times, insurance status and health literacy [14, 15]. Historically, in the UK, it was estimated that 95% of people with new Hepatitis B diagnoses were immigrants [14], who may require further support in attending appointments in a healthcare system they are less familiar with.

In response to some of the aforementioned barriers to non‐attendance, different modes of appointment delivery have been trialled. For example, Bowyer [16] reported on clinics delivered by specialist nurses in primary care (general practice) settings, but found that non‐attendance was higher than in secondary care settings (23% compared to 19%). When questioned, patients admitted they had not noticed that the setting had changed, despite receiving letters about it. In another study, authors found that virtual appointments had lower non‐attendance rates than face‐to‐face appointments [11]. Unfortunately, non‐attendance at appointments remains a complex challenge regardless of the care delivery approach.

A better understanding of the factors contributing to non‐attendance among people with Hepatitis B in secondary care could help improve service delivery and patient outcomes. Yet no reviews currently exist on this topic, suggesting a limited understanding of the availability, breadth and extent of knowledge in this area. This scoping review sought to address the research gap by synthesising current evidence on non‐attendance among people living with Hepatitis B to identify strategies that may enhance clinic attendance and continuity of care.

## 2 Aims and Review Question

The review aims are twofold:

1. To map the existing reasons influencing non‐attendance at Hepatitis B secondary care services.
2. To highlight evidence gaps and inform future research.

Considering these aims, this review asks, “What evidence exists on the reasons for non‐attendance at Hepatitis B secondary care services, and what interventions are reported to address this?”

## 3 Methods

This scoping review was conducted in accordance with the Joanna Briggs Institute (JBI) methodology [17] and reported using the Preferred Reporting Items for Systematic Reviews and Meta‐Analyses extension for Scoping Reviews (PRISMA‐ScR) guidelines [18]—see Data S1.

The protocol is registered and available in the Open Science Framework (https://osf.io/fu3tw/overview).

### 3.1 Inclusion Criteria

The inclusion criteria followed the ‘PCC’ (Population, Concept, Context) framework:

**Population (P):** Adults diagnosed with Hepatitis B in the UK.

**Concept (C):** Factors influencing attendance, non‐attendance and interventions to improve attendance of secondary care appointments.

**Context (C):** Secondary care services, such as hospital outpatient clinics or specialist liver services in the UK.

No date filters were used to ensure wide capture. Sources that did not fully meet the inclusion criteria but contained relevant data on Hepatitis B in the UK were included (e.g., sources including multiple countries or types of hepatitis), with only the relevant data extracted. Articles not available in English, grey literature and conference abstracts were excluded. While reviews were included in the search, none were identified.

### 3.2 Search Strategy

Searches were carried out in August 2025. Databases included CINAHL Ultimate (EBSCOhost), Medline (Ovid), EMBASE, Scopus and APA PsycINFO.

Query strings were developed by BG and EVS and included several MeSH terms. For transparency, the full electronic search strategy for one database (Medline) is presented:

Search terms: *Hepatitis B* (*exp Hepatitis B*/), *adults* (*exp Adult*/), *United Kingdom* (*UK.mp. or exp United Kingdom*/), *and attendance‐related terms including attendance* (*attendance.mp*.), *non‐attendance* (*No‐Show Patients*/*or non‐attendance.mp*.), *and participation* (*participation.mp. or Patient Participation*/).

Search terms were combined using Boolean operators. Population and context terms were combined using ‘AND’ (1 AND 2 AND 3), while attendance‐related concepts were grouped using ‘OR’ (4 OR 5 OR 6). Both sets were then combined (7 AND 8) to identify studies relevant to Hepatitis B and appointment attendance in the UK adult population.

To verify accuracy, two pre‐identified articles were selected as benchmarks for testing the search process. Further details are given in the protocol: https://osf.io/fu3tw/overview.

### 3.3 Study/Source of Evidence Selection

All search results were imported into Rayyan [19]. After duplicates were removed, B.G. and E.V.S. blindly screened the remaining articles using a two‐step process. This was done first by screening titles and abstracts, and secondly by screening full texts. Full‐text retrieval and conflict resolution were completed with a third researcher (N.G.).

### 3.4 Data Extraction

Data were extracted using a structured data charting form, which was developed and pilot‐tested on five articles by E.V.S. alongside B.G. One human extractor (B.G.) performed data extraction under the supervision of a second reviewer (E.V.S.). Any discrepancies were discussed and resolved with the wider team (N.G.).

Data were extracted for the following variables: full APA citation, year, country, study population (including age, sex and relevant clinical characteristics), health condition(s), study methodology, data collection methods, interventions used, analysis approach, research questions or aims, key findings related to attendance or barriers/facilitators, database in which the study was indexed.

The data extraction table is provided as Data S2.

### 3.5 Quality Appraisal

A quality appraisal of each included study was conducted using the Mixed Methods Appraisal Tool (MMAT) [20] to inform the interpretation of results and assess the methodological quality and potential sources of bias. This was conducted not to identify low‐quality research for exclusion but to help inform recommendations for future research. Authors E.V.S. and B.G. conducted the quality appraisal independently. The initial agreement rate was 82%, with disagreements in two sources resolved through discussion. Full details are presented as Data S3.

### 3.6 Analysis

Available evidence was synthesised to examine the factors influencing engagement with secondary care services among people living with Hepatitis B, as well as any strategies to improve attendance. Analysis consisted of descriptive statistics, followed by basic qualitative content analysis. Together, they revealed substantial insights and gaps.

## 4 Results

### 4.1 Characteristics and Quality of the Studies

**FIGURE 1:**
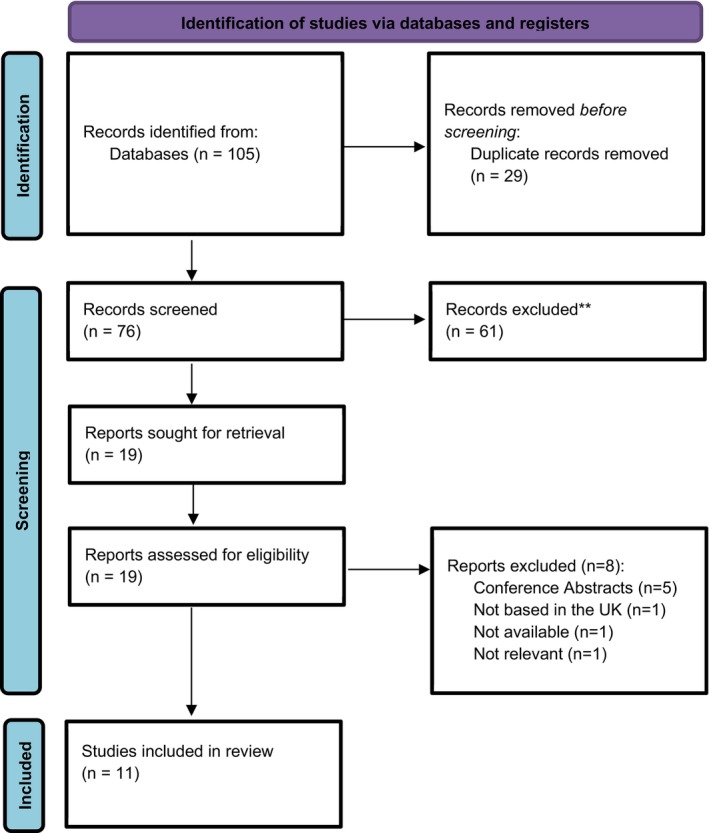
Flow diagram presenting the search and screening process; adhering to the Preferred Reporting Items for Systematic Reviews and Meta‐Analysis.

This scoping review identified only 11 relevant articles (See Figure 1), displaying a limited evidence base. Eight studies were excluded at the full‐text screening stage due to the following reasons: conference abstracts (*n* = 5), not based in the UK (*n* = 1), not available (*n* = 1) and not relevant (*n* = 1).

Sources tended to be quantitative (*n* = 8) in approach, with the remainder being qualitative (*n* = 2) or mixed‐methods (*n* = 1); leaving a sizable experiential gap in the evidence base (see Table 1).

**TABLE 1:** Data collection methods of the included sources (*N* = 11).

Sources	Interviews/Focus groups	Surveys/Questionnaires	Clinical testing/Screening	Database/Secondary data	Administrative/Case records
Baldry et al. [18]		X			
Eborall et al. [19]	X				
Parry et al. [20]			X	X	X
Sweeney et al. [21]	X	X			
Teague et al. [22]			X	X	
Barclay et al. [23]				X	
Kelly et al. [24]		X	X		
Hibbert et al. [25]				X	
Kelly et al. [26]	X	X	X		
Weaver et al. [27]	X		X		X
Williams et al. [28]				X	

Although research needed to be conducted in the UK to be eligible for inclusion, specific regions in which they were conducted were not always reported, limiting the ability to account for geographic variation in migrant populations, local healthcare access and regional differences in Hepatitis B prevalence, which are all critical for accurately interpreting findings and informing targeted public health interventions (see Table 2).

**TABLE 2:** Descriptive characteristics of the included sources (*N* = 11).

Characteristics	*N*
Methodology	
Quantitative	8
Qualitative	2
Mixed‐methods	1
Methods of data collection	
Other, for example, screening	8
Interviews/focus groups	2
Surveys/questionnaires	1
Sample size	
> 1000	6
100–1000	4
N/A (mathematical model)	1
Region reported	
UK	8
London	2
England	1

All studies were of either acceptable (*n* = 5) or excellent (*n* = 6) quality. However, none were rigorous randomised controlled trials meaning the effectiveness of potential solutions (i.e., interventions) remains insufficiently understood [21, 23, 25, 29, 30, 31]. Additionally, the overall depth of available evidence was limited and somewhat variable in quality [26, 30, 31] (See Table 3). Given that current understandings largely rely on quantitative research, potential nuances and the reasoning behind attendance or non‐attendance were also missed [22, 24]. Further, few used robust theoretical frameworks or clearly defined outcome measures, further restricting firm conclusions on the effectiveness of specific engagement strategies [21, 29, 30, 31].

**TABLE 3:** Quality appraisal.

Authors	Appraisal (/5)	Rating
Baldry et al. [19]	3	Acceptable
Eborall et al. [20]	5	Excellent
Parry et al. [21]	3	Acceptable
Sweeney et al. [22]	5	Excellent
Teague et al. [23]	3	Acceptable
Barclay et al. [24]	5	Excellent
Kelly et al. [25]	5	Excellent
Hibbert et al. [26]	5	Excellent
Kelly et al. [27]	4	Acceptable
Weaver et al. [28]	4	Acceptable
Williams et al. [29]	5	Excellent

### 4.2 Barriers to Attending Secondary Care Appointments

Across the 11 included studies, engagement with secondary care was shaped by a complex interaction of personal, cultural and structural factors [22, 24, 27, 29]. Key obstacles to attendance were: limited knowledge of Hepatitis B, stigmatisation around routes of transmission, competing life priorities, socioeconomic constraints (e.g., long work hours, limited/no sick pay, homelessness), language difficulties (e.g., English as a second language) and fragmented healthcare navigation pathways [21, 22, 23, 24, 25, 26, 27, 28, 29].

Specific challenges existed for migrant communities, such as fear that testing positive for an infectious disease (e.g., Hepatitis B) could negatively impact their visa or citizenship applications, leading to potential deportation or rejection [22]. People with poor health literacy were also reported to be at risk of non‐attendance, as the absence of obvious symptoms often leads people to believe they are healthy [24]. This is particularly noteworthy as Hepatitis B generally is asymptomatic, meaning many individuals seldom seek help, increasing the likelihood of complications. Medical jargon was further identified as inaccessible and confusing (e.g., believing a ‘positive’ diagnosis to be a good thing), which can decrease adherence to health‐promoting behaviours [21]. Cultural beliefs, such as fatalism, were also noted as deterrents [27]; though maintaining positive relationships and knowledge exchange with community and religious leaders (e.g., Imams, local councillors), could reduce this [29].

### 4.3 Interventions to Address Non‐Attendance

Among the data sources, interventions to combat non‐attendance tended to be highly localised and varied widely in scope, for example, reminder systems, culturally adapted education, community outreach, case‐management models [22, 24, 25, 27, 29, 30]. Financial incentives, such as vouchers, also appeared useful for capturing a more diverse ‘at‐risk’ population: those experiencing homelessness or poverty [29]. Additionally, transforming the current fragmented system into a ‘one‐stop’ model where all testing and assessment components can happen in a single visit could vastly reduce attrition [22, 29].

Community involvement and co‐production were well‐documented solutions to non‐adherence [22, 24, 27, 29]. These approaches were especially important for people from migrant and minority ethnic backgrounds, improving cultural relevance, acceptability and real‐world feasibility through culturally appropriate and acceptable interventions [22, 24, 27, 29]. Multicultural link workers and peer advocates who speak multiple languages and understand particular cultures were suggested to bridge gaps between patients and clinicians, often leading to increased understanding and reduced anxiety/hesitation to seek help [21, 27]. Additionally, the research pointed to partnerships between clinical teams, public health services and community organisations as also conducive to creating patient‐centred strategies that address both medical and social factors influencing engagement [22, 24, 27, 29].

## 5 Discussion

This scoping review provides insight into the reasons for non‐attendance at Hepatitis B secondary care appointments. Generally, reasons for non‐attendance were reported to revolve around knowledge, attitudes, competing priorities, socioeconomic constraints and systematic issues. But given the number of quantitative sources within the data set, the available literature falls short of delivering concrete evidence on the effectiveness of interventions that could inform sustainable and feasible strategies to addressing non‐attendance. Additionally, qualitative evidence was limited; thus, there was little exploration regarding nuances that could be key to promoting appointment attendance.

Identifying solutions for non‐attendance in Hepatitis B populations is essential, not only because it aligns with the WHO's Hepatitis B elimination goals [11], but also because increased attendance may reduce disparities in health outcomes and minimise future costs to the NHS, which is a priority for the 2025/6 year [32]. Urgency around Hepatitis B continues to grow with recent reports of England ‘falling short’ of meeting WHO's 2030 elimination goals—unless diagnosis and treatment are improved [4]. Although these domains rely on patient engagement, which is evidenced by this review as wavering [21, 22, 23, 24, 25, 26, 27, 28, 29].

Interestingly, broader disease variations, that is, Hepatitis A, lead to higher engagement rates than Hepatitis B, particularly around treatment [33] (vaccine). However, this may be linked to Hepatitis A's decreased stigma, due to [typically] being short‐term faecal‐oral transmission and its ‘blameless’ perception [33]. This differs from the transmission of Hepatitis B and C: blood/fluids, or blood‐to‐blood [34], which are often viewed as taboo for their associations with unprotected sex and intravenous drug use [35].

While Hepatitis C services also struggle with engagement, this is usually around prevention (e.g., lack of vaccine). Additionally, Hepatitis C's high curability and revolutionised treatments mean further success than Hepatitis B, which can only be suppressed through sustained self‐management and professional intervention [36]. Further, Hepatitis B requires a three‐dose vaccination schedule, compared to the two‐dose schedule for Hepatitis A, which may reduce the likelihood of patients completing the full vaccine series [37]. When compared to its A and C counterparts, Hepatitis B is falling behind in survival and treatment adherence rates [38], meaning that to meet the WHO's elimination targets [4], resolution must be sought within the next four years.

The findings of this scoping review suggest specific challenges related to supporting migrant populations in attending appointments, as well as cultural tension [22, 24, 27, 29]. Wider evidence consistently presents migrant populations from outside Europe and the USA to experience a higher prevalence of Hepatitis B, due to their countries of origin often having higher infection rates [39]. In the UK, between 80% and 90% of people with newly diagnosed Hepatitis B were born in countries with intermediate or high prevalence [40] such as China or Pakistan [41, 42, 43, 44]; indicating a clear need for Hepatitis B interventions that are tailored and sensitive to differing cultural needs. Transferable learning from other stigmatised health conditions, for example, COPD, or mental health [45, 46], suggests that culturally informed practice is critical in developing acceptable, scalable and sustainable models of care that enhance long‐term engagement among these communities. Particularly as ethnic minorities often face additional barriers to help‐seeking due to complexities between cultural misconceptions, systemic issues, lower health literacy and stigmatisation [47, 48].

Research consistently reports that migrant and ethnic minority populations face both system and provider‐level obstacles within the NHS, including accessing culturally appropriate services and uncertainty around eligibility [49]. Critically, these challenges are not UK exclusive [50] reflecting a global pattern in which the same vulnerable populations are repeatedly failed across differing healthcare systems. In the context of HBV, where these groups already carry the greatest disease burden [47, 48] this constitutes a preventable and recurring inequity.

Evidence now consistently shows that cultural and contextual factors significantly shape engagement with health interventions [45], meaning cultural obstacles must be considered in future strategies. This is particularly relevant for South Asian communities, the UK's largest ethnic minority group [51, 52], who—alongside Black, Black British, Caribbean and African UK residents—experience more than double the overall incidence of Hepatitis B cases [4, 41, 53].

The 2024 WHO hepatitis B guidelines [54] also recognise limited access to HBV DNA testing and the complex multi‐visit diagnostic pathways as significant obstacles within HBV care—as early as diagnosis. Encouragingly, emerging technologies may address some of these barriers. For instance, point‐of‐care HBV DNA testing represents a promising emerging solution, showing same‐day results and high rates of testing uptake and treatment initiation among eligible individuals [55]. A simple fingerstick blood test may also streamline care pathways and improve patient engagement [56], particularly among the underserved populations identified in this review.

Going forward, the need for additional research is clear. Future quantitative Hepatitis B research may incorporate stronger methodological designs, including well‐defined engagement outcomes and appropriate comparison groups that measure the impact of interventions on attendance, adherence and long‐term clinical outcomes. Additionally, implementation science frameworks may enhance the ability to evaluate feasibility, sustainability and scalability within routine clinical pathways, which were flagged as problematic for attendance [21, 29]. Meanwhile, future qualitative investigation in this area may seek further nuance on the reasoning behind attendance or non‐attendance, as well as support the co‐production of strategies to help people living with Hepatitis B attend their appointments.

This scoping review is limited by several methodological constraints. First, data extraction was completed by a single reviewer (B.G.), which may introduce bias. Although provided supervision may have mitigated the extent (E.V.S.). Second, this review captured few studies (*n* = 11), meaning inclusion of grey literature could have been fruitful—particularly reports, guidelines, or unpublished studies. However, relevant reports, for example, those by the WHO, have been incorporated into the discussion. Finally, this was a UK‐based project, which may limit generalisability overseas. That said, to the best of our knowledge, this is the only scoping review of Hepatitis B attendance in UK secondary care services, indicating a novel contribution.

## 6 Conclusion

This scoping review mapped the available evidence of non‐attendance among people with Hepatitis B in UK secondary care. Findings reveal that attendance is generally influenced by personal, social and systemic factors, which may interact with one another. Current interventions vary widely in scope, but tend to be highly localised, for example, reminder systems, culturally adapted education, community outreach and case‐management models. Findings infer that interventions that address multiple factors, particularly stigma and cultural obstacles, may improve engagement—especially within minority communities who are most at‐risk of infection. Although it is important to note that people with Hepatitis B are not a monolith, therefore, further research is needed to co‐design and evaluate interventions that are sensitive to the needs of each affected community/person.

## Author Contributions

Supervision: Norina Gasteiger, Emma Victoria Shiel and Arpana Verma. Conceptualisation: Bini George and Arpana Verma. Design: Emma Victoria Shiel, Bini George, Arpana Verma and Norina Gasteiger. Searches, screening, extraction, quality appraisals and analysis: Bini George and Emma Victoria Shiel. Writing – original draft: Emma Victoria Shiel and Bini George. Writing – review and editing: Emma Victoria Shiel, Bini George, Arpana Verma and Norina Gasteiger.

## Funding

This research study was funded by the NIHR Applied Research Collaboration (Grant award: NIHR200174). The views expressed in this publication are those of the authors and not necessarily those of the National Institute for Health and Care Research or the Department of Health and Social Care.

## Conflicts of Interest

The authors declare no conflicts of interest.

## Supporting information


**Data S1:** Preferred Reporting Items for Systematic reviews and Meta‐Analyses extension for Scoping Reviews (PRISMA‐ScR) checklist.

**Data S2:** Data extraction table, characteristics of included studies.

**Data S3:** Mixed Methods Appraisal Tool.

## Data Availability

The data that supports the findings of this study are available in the Supporting Information of this article.

## References

[1] World Health Organization , “Hepatitis B,” (2025), https://www.who.int/news‐room/fact‐sheets/detail/hepatitis‐bwho.int.

[2] W. J. Jeng , G. V. Papatheodoridis , and A. S. F. Lok , “Hepatitis B,” Lancet 401, no. 10381 (2023): 1039–1052. 36774930 10.1016/S0140-6736(22)01468-4

[3] World Health Organization , Global Health Sector Strategy on Viral Hepatitis, 2016–2021: Towards Ending Viral Hepatitis (World Health Organization, 2016), https://www.who.int/publications/i/item/WHO‐HIV‐2016.06.

[4] UK Health Security Agency , “Hepatitis B in England 2025,” (2025), https://www.gov.uk/government/publications/hepatitis‐b‐in‐england/hepatitis‐b‐in‐england‐2025.

[5] B. J. McMahon , “Meeting the WHO and US Goals to Eliminate Hepatitis B Infection by 2030: Opportunities and Challenges,” Clinical Liver Disease 12, no. 1 (2018): 29–32. 30988906 10.1002/cld.733 PMC6385905

[6] L. F. Dantas , J. L. Fleck , F. L. C. Oliveira , and S. Hamacher , “No‐Shows in Appointment Scheduling–a Systematic Literature Review,” Health Policy 122, no. 4 (2018): 412–421. 29482948 10.1016/j.healthpol.2018.02.002

[7] D. L. Wolff , F. B. Waldorff , C. von Plessen , et al., “Rate and Predictors for Non‐Attendance of Patients Undergoing Hospital Outpatient Treatment for Chronic Diseases: A Register‐Based Cohort Study,” BMC Health Services Research 19, no. 1 (2019): 386. 31200720 10.1186/s12913-019-4208-9 PMC6570866

[8] L. G. Pérez , S. Marciano , and D. H. Giunta , “Temporal Trends in the Non‐Attendance to Scheduled Outpatient Appointments,” AJE Advances: Research in Epidemiology 1, no. 3 (2025): uuaf021.

[9] I. B. Afolabi , A. B. Aremu , M. J. P. Koryom , A. Buh , S. A. Bainbridge , and K. P. Phillips , “Predictors of Medication Non‐Adherence Among Hepatitis B Patients in South Sudan: A Health‐Facility‐Based Cross‐Sectional Study,” Patient Preference and Adherence 19 (2025): 981–996. 40230460 10.2147/PPA.S514283 PMC11995916

[10] National Institute for Health and Care Excellence [NICE] , “Quality Statement 5: Personalised Care Plan,” (2014), https://www.nice.org.uk/guidance/qs65/chapter/quality‐statement‐5‐personalised‐care‐plan.

[11] J. Sun , A. Al‐Hasani , J. Antony , et al., “P52 Factors Determining Outpatient Clinic Attendance in Patients With Chronic Hepatitis B Infection,” Gut 73 (2024): A80.

[12] N. L. Allard , J. H. MacLachlan , A. Dev , et al., “Adherence in Chronic Hepatitis B: Associations Between Medication Possession Ratio and Adverse Viral Outcomes,” BMC Gastroenterology 20, no. 1 (2020): 140. 32381025 10.1186/s12876-020-01219-w PMC7203797

[13] S. Ahmad , S. Paracha , R. Ali , et al., “Nonadherence to Antiviral Therapy Leading to Severe Hepatic Decompensation in a Patient With Chronic Hepatitis B,” ACG Case Reports Journal 13, no. 2 (2026): e01999. 41696665 10.14309/crj.0000000000001999 PMC12900210

[14] L. B. Rustam , M. Vander Weg , E. Chrischilles , and T. Tanaka , “Sociodemographic and Clinical Factors Associated With Nonattendance at the Hepatology Clinic,” Digestive Diseases and Sciences 68, no. 6 (2023): 2398–2405. 37106247 10.1007/s10620-023-07951-z

[15] M. Alturbag , “Factors and Reasons Associated With Appointment Non‐Attendance in Hospitals: A Narrative Review,” Cureus 16, no. 4 (2024): e58594. 38765331 10.7759/cureus.58594 PMC11102763

[16] T. Bowyer , “A Nurse‐Led Community Hepatitis B Clinic,” Nursing Times 110, no. 7 (2014): 18–19. 24672909

[17] M. D. J. Peters , C. Godfrey , P. McInerney , C. B. Soares , H. Khalil , and D. Parker , “Methodology for JBI Scoping Reviews,” in The Joanna Briggs Institute Reviewers' Manual 2015, ed. E. Aromataris (Joanna Briggs Institute, 2015), 3–24.

[18] A. C. Tricco , E. Lillie , W. Zarin , et al., “PRISMA Extension for Scoping Reviews (PRISMA‐ScR): Checklist and Explanation,” Annals of Internal Medicine 169, no. 7 (2018): 467–473. 30178033 10.7326/M18-0850

[19] M. Ouzzani , H. Hammady , Z. Fedorowicz , and A. Elmagarmid , “Rayyan—A Web and Mobile App for Systematic Reviews,” Systematic Reviews 5, no. 1 (2016): 210. 27919275 10.1186/s13643-016-0384-4 PMC5139140

[20] Q. N. Hong , S. Fàbregues , G. Bartlett , et al., “The Mixed Methods Appraisal Tool (MMAT) Version 2018 for Information Professionals and Researchers,” Education for Information 34, no. 4 (2018): 285–291.

[21] G. Baldry , D. Phillips , R. Wilkie , et al., “Factors Associated With Human Papillomavirus, Hepatitis A, Hepatitis B and Mpox Vaccination Uptake Among Gay, Bisexual and Other Men Who Have Sex With Men in the UK–Findings From the Large Community‐Based RiiSH‐Mpox Survey,” International Journal of STD & AIDS 35, no. 12 (2024): 963–981. 39163149 10.1177/09564624241273778

[22] H. Eborall , F. Wobi , K. Ellis , et al., “Integrated Screening of Migrants for Multiple Infectious Diseases: Qualitative Study of a City‐Wide Programme,” EClinicalMedicine 21 (2020): 100315. 32322806 10.1016/j.eclinm.2020.100315 PMC7170938

[23] S. Parry , N. Bundle , S. Ullah , et al., “Implementing Routine Blood‐Borne Virus Testing for HCV, HBV and HIV at a London Emergency Department–Uncovering the Iceberg?,” Epidemiology and Infection 146, no. 8 (2018): 1026–1035. 29661260 10.1017/S0950268818000870 PMC9184937

[24] L. Sweeney , J. A. Owiti , A. Beharry , et al., “Informing the Design of a National Screening and Treatment Programme for Chronic Viral Hepatitis in Primary Care: Qualitative Study of At‐Risk Immigrant Communities and Healthcare Professionals,” BMC Health Services Research 15, no. 1 (2015): 97. 25890125 10.1186/s12913-015-0746-y PMC4372168

[25] A. Teague , J. Zeng , T. Haque , D. Macdonald , and K. Bryce , “Engagement of Patients With Viral Hepatitis Diagnosed by Opt‐Out Universal Emergency Department Testing,” Journal of Hepatology 78 (2023): S872–S873.

[26] S. T. Barclay , S. Cameron , P. R. Mills , et al., “The Changing Face of Hepatitis B in Greater Glasgow: Epidemiological Trends 1993–2007,” Scottish Medical Journal 55, no. 3 (2010): 4–7. 20795508 10.1258/rsmsmj.55.3.4

[27] C. Kelly , M. Pericleous , A. Ahmed , et al., “Improving Uptake of Hepatitis B and Hepatitis C Testing in South Asian Migrants in Community and Faith Settings Using Educational Interventions—A Prospective Descriptive Study,” International Journal of Infectious Diseases 100 (2020): 264–272. 32861830 10.1016/j.ijid.2020.08.059

[28] M. Hibbert , R. Simmons , N. Ratna , et al., “Retrospective Cohort Study Assessing Coverage, Uptake and Associations With Hepatitis B Vaccination Among Females Who Engage in Sex Work Attending Sexual Health Services in England Between 2015 and 2019,” Sexually Transmitted Infections 99, no. 7 (2023): 497–501. 37550014 10.1136/sextrans-2023-055845 PMC10715460

[29] C. Kelly , S. Mathew , M. Petrova , et al., “Exploring Awareness and Prevalence of Chronic Viral Hepatitis in a UK Based Nepali Population–Lessons Learned for Future Models in Engaging Migrant Communities,” Clinical Medicine 23, no. 6 (2023): 563–570. 38065610 10.7861/clinmed.2022-0356 PMC11046676

[30] T. Weaver , N. Metrebian , J. Hellier , et al., “Use of Contingency Management Incentives to Improve Completion of Hepatitis B Vaccination in People Undergoing Treatment for Heroin Dependence: A Cluster Randomised Trial,” Lancet 384, no. 9938 (2014): 153–163. 24725468 10.1016/S0140-6736(14)60196-3

[31] J. R. Williams , D. J. Nokes , and R. M. Anderson , “Targeted Hepatitis B Vaccination—A Cost Effective Immunisation Strategy for the UK?,” Journal of Epidemiology & Community Health 50, no. 6 (1996): 667–673. 9039387 10.1136/jech.50.6.667 PMC1060385

[32] NHS England , “2025/26 Priorities and Operational Planning Guidance,” (2025), https://www.england.nhs.uk/long‐read/2025‐26‐priorities‐and‐operational‐planning‐guidance/.

[33] H. M. Lekas , K. Siegel , and J. Leider , “Felt and Enacted Stigma Among HIV/HCV‐Coinfected Adults: The Impact of Stigma Layering,” Qualitative Health Research 21, no. 9 (2011): 1205–1219. 21498828 10.1177/1049732311405684 PMC4323279

[34] National Health Service [NHS] , “Hepatitis,” (2025), https://www.nhs.uk/conditions/hepatitis/.

[35] L. Huang , X. Chen , and Z. Wang , “Total Burden of Hepatitis B and C Attributed to Injecting Drug Use in 204 Countries and Territories From 1990 to 2021: Analyses Based on the Global Burden of Disease Study 2021,” International Journal of Infectious Diseases 150 (2025): 107293. 39505253 10.1016/j.ijid.2024.107293

[36] Hepatitis B Foundation , “What's the Difference: Hepatitis B vs Hepatitis C?,” (2019), https://www.hepb.org/blog/whats‐the‐difference‐hepatitis‐b‐vs‐hepatitis‐c/.

[37] K. Bruxvoort , J. Slezak , R. Huang , et al., “286. Hepatitis B Vaccine Compliance: Comparing 2‐Dose and 3‐Dose Vaccines,” Open Forum Infectious Diseases 6, no. 2 (2019): S156.

[38] M. Kumar , S. Pahuja , P. Khare , and A. Kumar , “Current Challenges and Future Perspectives of Diagnosis of Hepatitis B Virus,” Diagnostics 13, no. 3 (2023): 368. 36766473 10.3390/diagnostics13030368 PMC9914745

[39] UK Health Security Agency , Hepatitis B: Migrant Health Guide (GOV UK, 2025), https://www.gov.uk/guidance/hepatitis‐b‐migrant‐health‐guide.

[40] World Health Organization [WHO] , Guidelines for the Prevention, Diagnosis, Care and Treatment for People With Chronic Hepatitis B Infection (World Health Organization, 2024), https://iris.who.int/handle/10665/376353/. 40424433

[41] S. Hahné , M. Ramsay , K. Balogun , W. J. Edmunds , and P. Mortimer , “Incidence and Routes of Transmission of Hepatitis B Virus in England and Wales, 1995–2000: Implications for Immunisation Policy,” Journal of Clinical Virology 29, no. 4 (2004): 211–220. 15018847 10.1016/j.jcv.2003.09.016

[42] I. Evlampidou , M. Hickman , C. Irish , et al., “Low Hepatitis B Testing Among Migrants: A Cross‐Sectional Study in a UK City,” British Journal of General Practice 66 (2016): e382–e391. 10.3399/bjgp16X684817 PMC4871303 27025556

[43] A. Cochrane , I. Evlampidou , C. Irish , S. M. Ingle , and M. Hickman , “Hepatitis B Infection Prevalence by Country of Birth in Migrant Populations in a Large UK City,” Journal of Clinical Virology 68 (2015): 79–82. 26071342 10.1016/j.jcv.2015.05.009

[44] N. K. Martin , P. Vickerman , S. Khakoo , et al., “Chronic Hepatitis B Virus Case‐Finding in UK Populations Born Abroad in Intermediate or High Endemicity Countries: An Economic Evaluation,” BMJ Open 9, no. 6 (2019): e030183. 10.1136/bmjopen-2019-030183 PMC6609059 31256040

[45] R. E. Otaigboria , “Cultural Models of Illness and Health Communication Strategies Improving Healthcare Access and Equity for Immigrant Patients' Populations,” GSC Biological and Pharmaceutical Sciences 29, no. 3 (2024): 390–410.

[46] E. V. Shiel , J. Miah , T. Chattopadhyay , et al., “Understanding Barriers and Facilitators to Education and Rehabilitation Interventions for South Asian People With Long‐Term Conditions: A Systematic Review and Meta‐Ethnography,” BMJ Open 16, no. 1 (2026): e106694. 10.1136/bmjopen-2025-106694 PMC12815045 41529880

[47] E. Martyn , S. Eisen , N. Longley , et al., “The Forgotten People: Hepatitis B Virus (HBV) Infection as a Priority for the Inclusion Health Agenda,” eLife 12 (2023): e81070. 36757862 10.7554/eLife.81070 PMC9910830

[48] E. Stepanova , S. Croke , G. Yu , O. Bífárìn , M. Panagioti , and Y. Fu , “‘I Am Not a Priority’: Ethnic Minority Experiences of Navigating Mental Health Support and the Need for Culturally Sensitive Services During and Beyond the Pandemic,” BMJ Mental Health 28, no. 1 (2025): e301481. 10.1136/bmjment-2024-301481 PMC12083255 40280628

[49] L. Zibrik , A. Huang , V. Wong , et al., “Let's Talk About B: Barriers to Hepatitis B Screening and Vaccination Among Asian and South Asian Immigrants in British Columbia,” Journal of Racial and Ethnic Health Disparities 5, no. 6 (2018): 1337–1345. 29557047 10.1007/s40615-018-0483-0

[50] M. Jackson , Y. Ibrahim , C. Freeland , S. Jacob , B. Zovich , and C. Cohen , “Barriers to Accessing Hepatitis B Medication: A Qualitative Study From the USA and Canada,” BMJ Open 14, no. 5 (2024): e080658. 10.1136/bmjopen-2023-080658 PMC11110584 38772585

[51] G. Iqbal , M. R. Johnson , A. Szczepura , S. Wilson , A. Gumber , and J. A. Dunn , “UK Ethnicity Data Collection for Healthcare Statistics: The South Asian Perspective,” BMC Public Health 12, no. 1 (2012): 243. 22452827 10.1186/1471-2458-12-243 PMC3339513

[52] UK Government , “Population of England and Wales. Ethnicity Facts and Figures,” (2024), https://www.ethnicity‐facts‐figures.service.gov.uk/uk‐population‐by‐ethnicity/national‐and‐regional‐populations/population‐of‐england‐and‐wales/latest/.

[53] R. Simmons , R. Harris , A. G. Lim , et al., “Estimating Prevalence and Number of People With Chronic Hepatitis B: A Multiplier Method Based on Public Health Surveillance Data in UK (2015–2021),” Journal of Viral Hepatitis 32, no. 4 (2025): e14019. 39412144 10.1111/jvh.14019 PMC11899512

[54] World Health Organization , “Guidelines for the Prevention, Diagnosis, Care and Treatment for People With Chronic Hepatitis B Infection,” (2024), https://www.who.int/publications/i/item/9789240090903. 40424433

[55] S. Gu , Y. Tao , C. Fan , et al., “Impact of Hepatitis B Virus Point‐Of‐Care DNA Viral Load Testing Compared With Laboratory‐Based Standard‐Of‐Care Approaches on Uptake of HBV Viral Load Testing, Treatment, and Turnaround Times: A Systematic Review and Meta‐Analysis,” Open Forum Infectious Diseases 11, no. 9 (2024): ofae483. 39296343 10.1093/ofid/ofae483 PMC11409893

[56] B. Hajarizadeh , J. George , M. T. Levy , et al., “Evaluation of Fingerstick Blood Point‐Of‐Care Testing of Hepatitis B DNA for Enhanced Hepatitis B Treatment Decision Making: A Diagnostic Accuracy Study,” Journal of Clinical Microbiology 64 (2026): e0140525. 41556653 10.1128/jcm.01405-25 PMC12892972

